# Epidemiological analysis to identify predictors of X-linked hypophosphatemia (XLH) diagnosis in an Italian pediatric population: the EPIX project

**DOI:** 10.1007/s12020-024-03793-5

**Published:** 2024-04-09

**Authors:** Salvatore Crisafulli, Ylenia Ingrasciotta, Giacomo Vitturi, Andrea Fontana, Luca L’Abbate, Ylenia Alessi, Francesco Ferraù, Luigi Cantarutti, Debora Lazzerini, Salvatore Cannavò, Gianluca Trifirò

**Affiliations:** 1 Department of Medicine, University of Verona, Verona, Italy; 2 Department of Diagnostics and Public Health, University of Verona, Verona, Italy; 3 Unit of Biostatistics, IRCCS Casa Sollievo della Sofferenza, Foggia, Italy; 4 Department of Biomedical and Dental Sciences and Morphofunctional Imaging, University of Messina, Messina, Italy; 5 Department of Human Pathology of Adulthood and Childhood “G. Barresi” DETEV, University of Messina, Messina, Italy; 6 Società Servizi Telematici – Pedianet Project, Padova, Italy; 7 Medical Affairs, Kyowa Kyrin, Milano, Italy

**Keywords:** X-linked hypophosphatemia, Epidemiology, Rare diseases, Machine learning

## Abstract

**Purpose:**

X-linked hypophosphatemia (XLH) is a rare multi-systemic disease characterized by low plasma phosphate levels. The aim of this study was to investigate the annual XLH prevalence and internally evaluate predictive algorithms’ application performance for the early diagnosis of XLH.

**Methods:**

The PediaNet database, containing data on more than 400,000 children aged up to 14 years, was used to identify a cohort of XLH patients, which were matched with up to 10 controls by date of birth and gender. The annual prevalence of XLH cases per 100,000 patients registered in PediaNet database was estimated. To identify possible predictors associated with XLH diagnosis, a logistic regression model and two machine learning algorithms were applied. Predictive analyses were separately carried out including patients with at least 1 or 2 years of database history in PediaNet.

**Results:**

Among 431,021 patients registered in the PediaNet database between 2007–2020, a total of 12 cases were identified with a mean annual prevalence of 1.78 cases per 100,000 patients registered in PediaNet database. Overall, 8 cases and 60 matched controls were included in the analysis. The random forest algorithm achieved the highest area under the receiver operating characteristic curve (AUC) value both in the one-year prior ID (AUC = 0.99, 95% CI = 0.99–1.00) and the two-year prior ID (AUC = 1.00, 95% CI = 1.00–1.00) analysis. Overall, the XLH predictors selected by the three predictive methods were: the number of vitamin D prescriptions, the number of recorded diagnoses of acute respiratory infections, the number of prescriptions of antihistamine for systemic use, the number of prescriptions of X-ray of the lower limbs and pelvis and the number of allergology visits.

**Conclusion:**

Findings showed that data-driven machine learning models may play a prominent role for the prediction of the diagnosis of rare diseases such as XLH.

## Background

X-linked hypophosphatemic rickets, also known as X-Linked Hypophosphatemia (XLH), is a rare, hereditary, chronic, and progressive skeletal disease characterized by low plasma phosphate levels. XLH is caused by mutations in the *PHEX* gene, which is involved in regulating phosphate levels in the body [1]. Mutations in this gene result in increased concentrations of fibroblast growth factor 23 (FGF23), a protein that regulates renal phosphate reabsorption. In turn, high levels of FGF23 reduce the amount of phosphate that is reabsorbed by the kidneys, leading to the excessive elimination of phosphate in the urine and thus resulting in hypophosphatemia and symptoms related to this disease [2, 3].

The European prevalence of XLH reported in the literature ranges between 1.7 and 4.8 per 100,000 inhabitants [4, 5]. Due to the localization of the *PHEX* gene on the X chromosome, a woman with XLH has a 50% chance of genetically transferring the condition to each of her children, while a man with XLH will pass it on to all his daughters, but none of his sons. In some cases, this disease is not hereditary, but it can be related to a new spontaneous mutation (de novo) in the *PHEX* gene. XLH is usually diagnosed in childhood, based on a physical exam, blood tests, instrumental investigations (X-rays) and family history [6, 7]. If an early genetic analysis is not available, the disease is diagnosed on the basis of low phosphate levels, phosphaturia, presence of inadequate growth rate, noticeable bowing of the legs and/or other skeletal abnormalities, and eventually high levels of FGF23 in the blood; lack of phosphate levels’ response to treatment with oral vitamin D can be used to exclude a diagnosis of nutritional rickets. A genetic test identifying a mutation in the *PHEX* gene can confirm the diagnosis of XLH [2, 8, 9].

The course of the disease varies according to its severity and the onset of its complications. In children, XLH can lead to rickets, deformity of the lower limbs, overweight or obesity [10], delayed growth and inadequate final height [10]. In adulthood, some XLH patients may show minor clinical manifestations, while others may experience persistent issues and complications [11, 12]. Because XLH is a progressive, lifelong disease, recent evidence has suggested a likelihood of new deficits not previously seen in childhood [13, 14].

Unlike other types of rickets, XLH cannot be treated by only adjusting vitamin D levels [6]. Phosphate supplements are generally required, and they are typically associated with high doses of calcitriol. In children, treatment is usually started at the time of diagnosis and continued until the bones stop growing [12]. Depending on the symptomatology and severity of the disease, other treatment strategies may include somatotropin to promote growth in children, corrective surgery to reduce varus/valgus bending of the legs, treatment to correct abnormalities of the skull (e.g., craniosynostosis) and dental interventions for the treatment of teeth and gum problems [6].

In September 2019, the Italian Medicines Agency authorized the use of burosumab for the treatment of XLH with radiographic evidence of bone disease, in children aged 1 year and over and in adolescents with growing skeleton [15]. Burosumab is a fully human IgG1 monoclonal antibody that inhibits excess fibroblast growth factor 23 (FGF23) activity, with the aim of improving phosphate levels and bone mineralization [16]. Randomized clinical trials confirmed that switching from conventional therapy (calcitriol or alfacacidol, in combination with high doses of phosphate salts) to burosumab, especially early in life, resulted in rickets improvement [17, 18]. In March 2023 burosumab indication was extended to adults [19].

XLH epidemiological studies are extremely limited and, as in many rare diseases, XLH is frequently underdiagnosed or diagnosed late [20]. Generally, the diagnostic delay for rare diseases is around 4.8 years, and patients generally encounter an average of 7 different specialists before they receive the correct diagnosis [21]. Since this delay has a serious impact on patient’s quality of life, earlier identification of the disease is essential, not only for patient follow-up, but also for research purposes. For this reason, it is important to develop strategies facilitating the early diagnosis of XLH and the timely implementation of appropriate treatments to reduce the risk of complications related to progression of the disease. The aim of this study was to detect a validated set of diagnosis predictors, associated with the presence of the disease in the PediaNet database population, that could help family pediatricians (FPs) and healthcare professional to anticipate the XLH diagnosis.

## Methods

### Data source

This study is a non-interventional retrospective epidemiologic investigation based on existing data in the Italian pediatric database PediaNet, a pediatric general practice research database, including more than 300 FPs distributed throughout Italy collecting pseudonymised and computerized data during their daily practice. As of December 2020, PediaNet database included data related to 431,021 children aged up to 14 years, cared for by FPs involved in the PediaNet Network. This dataset includes data concerning demographics, lifestyle, parents’ socioeconomic status, weight, and height measurements (Supplementary Table 1). PediaNet database has been shown to provide accurate and reliable information, especially on patient’s diagnosis, for pharmacoepidemiology research, as documented elsewhere [22–24].

Diagnoses were recorded in free text or coded during routine clinical practice through the International Classification of Diseases - 9^th^ revision – clinical modification (ICD-9-CM). Drug prescriptions, including also over the counter (OTC) drugs, were registered using the Anatomical Therapeutic Chemical (ATC) Classification System and the Authorization Marketing Code (AIC). In addition, information on any vaccines that have been administered to children was available. Additional information available in PediaNet included reasons for healthcare services co-payment exemptions and requests for specialist visits and diagnostic tests.

Study primary endpoint was the calculation of the annual prevalence of XLH cases in the Pedianet database, while secondary endpoints were the evaluation of diagnosis predictors associated with the presence of the disease in the study population and internal evaluation of predictive diagnostic algorithms’ application performance.

### Study population

All potential XLH cases were identified from January 1, 2007 to December 31, 2020 through specific algorithms based on the presence of signs and symptoms of XLH. Cases already diagnosed with XLH were identified using: (i) the specific co-payment exemption code (RC0170: *resistant vitamin D hypophosphatemic rickets*) and/or (ii) clinical diagnosis recorded by the FPs (ICD-9 code 275.3: *Disorders of phosphorus metabolism*). For each identified XLH case, the date of the first occurrence of any XLH specific drug prescription (i.e., *calcitriol, ATC: A11CC04*), co-payment exemption or diagnosis recorded by the FPs (as free text or through the specific ICD-9-CM code) from PediaNet data sources, was defined as the “index date” (ID). Cases were matched with up to 10 controls by date of birth and gender. For each paired control, the same ID of the corresponding matched case was assigned. Controls with no registration in PediaNet prior to the ID of the corresponding matched case were excluded from the matching set. Cases and controls were included in the analyses only if their ID was between the date of first registration in the database and the date of the last information available.

### Data analysis

The annual prevalence of XLH cases per 100,000 patients registered in PediaNet was estimated by dividing the number of cases identified in each calendar year in the period between 2007 and 2020 by the total annual number of patients registered in PediaNet database. The 95% confidence interval (CI) of the XLH prevalence was estimated using the Clopper-Pearson exact method, based on the cumulative probabilities of the binomial distribution.

All XLH cases and matched controls were identified and characterized at the ID in terms of physical characteristics, XLH-related comorbidities, as reported in the literature [25–27] and evaluated any time prior to the ID, and number of drug prescriptions evaluated within one year prior the ID.

An univariable analysis was performed to assess the association between each candidate predictor and the presence of XLH. In particular, concerning dichotomous predictors (i.e., presence of comorbidities), exact conditional logistic regression models were performed, and associations were reported as odds ratios (cases vs. controls) along with 95% CIs. For all the other predictors, reported as counts (e.g., number of drugs or visits/exams prescriptions), over dispersed Poisson models were performed and associations were reported as mean ratios (cases vs. controls), along with their 95% CIs. In both cases, all *p*-values were corrected for multiple testing following the Bonferroni method. Only statistically significant associations (i.e., corrected *p*-values < 0.05) were shown in the results.

The optimal combination of all the predictors which, together, were significantly associated with the presence of XLH, was assessed by a cross-validated multivariable conditional logistic regression with the Least Absolute Shrinkage and Selection Operator (LASSO) penalty, which yielded a classification rule based on the individual linear weighted combination of included predictors, and by two different machine learning algorithms: (i) *the classification tree*, built using the Recursive PArtitioning and Regression Tree (RPART) algorithm, which define patients groups at different risk of XLH diagnosis and (ii) *the “Random Forest”*, an ensemble of classification trees which returned as output a ranking of all internally validated predictors from the most to the less important in terms of variable importance (i.e., strength of association with the diagnosis of the disease).

The discriminatory accuracy of each proposed method was assessed by the Area Under the Receiver Operator Characteristic (ROC) Curve (AUC) computed on the estimated individual probabilities of having XLH, along with its 95% CIs computed using the DeLong method. Moreover, diagnostic, and prognostic accuracy, i.e., sensitivity (SE), specificity (SP), F-score, positive and negative predictive value (PPV and NPV, respectively) were estimated at the threshold of the individual probabilities that reached the highest Youden Index in the ROC curve.

All analyses were carried out separately for patients with at least one-year of database history prior to ID and patients with at least two years of database history prior to ID.

A *p* value of <0.05 denotes statistical significance. All statistical analyses were carried out using the R Foundation for Statistical Computing software (ver. 4.0, packages: “clogitLasso”, “party”, “rpart”, “ranger”,“pROC”) and SAS Software, Release 9.4 (SAS Institute, Cary, NC, USA).

## Results

**Fig. 1:**
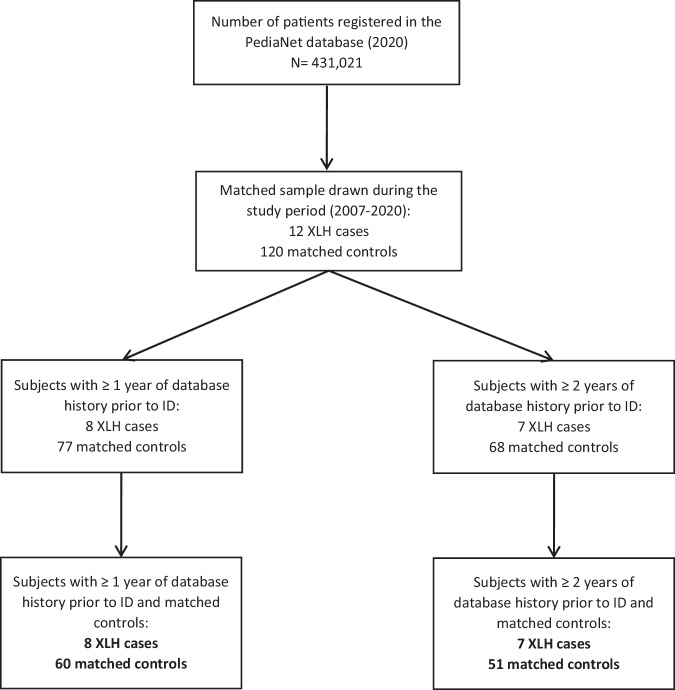
Flow-chart of cases and controls identified in PediaNet with at least one or two years of database history prior to the index date, respectively. ID Index Date, XLH X-linked hypophosphataemia

Overall, 431,021 patients were registered in the PediaNet database in the period between 2007 and 2020. Among them, a total of 12 XLH cases were identified through the specific co-payment exemption code and/or the clinical diagnosis recorded by the FPs. Since 4 XLH cases had less than one year of database history, only 8 cases were included in the one-year prior to ID analysis. Similarly, one more XLH case with less than 2 years of database history was not included in the two-years prior ID analysis (Fig. 1).

**Fig. 2:**
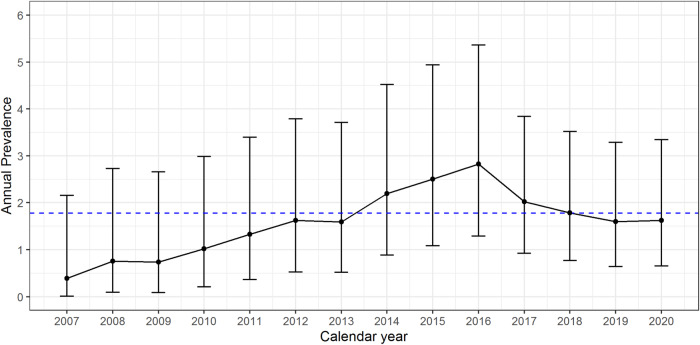
Plot of the annual X-linked hypophosphataemia prevalence per 100,000 individuals in the period 2007–2020. The dashed blue line represents the average annual prevalence

The mean XLH annual prevalence was 1.78 cases per 100,000 patients registered in PediaNet database, with an increasing trend from 2007 to 2016, followed by a slight decrease from 2016 to 2020 (Fig. 2).

The 8 XLH cases included in the one-year prior to ID analysis were matched to 60 controls. The age of XLH cases at ID ranged between 1 and 13 years, with a median age of 8.3 (IQR: 3.7–10.9) years and 75% of them were females (Table 1). Data on physical characteristics were not recorded in PediaNet for all the subjects included in the analysis, but no noticeable differences between cases and controls were observed. XLH-related comorbidities were recorded only for few XLH cases and the most reported were asthma (25.0 vs 11.7% among controls), fractures (12.5 vs 3.3% among controls), headache (12.5 vs 3.3% among controls) and acquired deformities of limbs (12.5 vs 3.3% among controls). The median number of individual drug prescriptions was higher for cases [3 (IQR: 1.5–9.0)] than for controls [1 (IQR: 0.0–4.0)]. The evaluation of some parameters attributable to XLH, especially biochemical signs, was limited by the amount of missing information (Supplementary Table 1).

**Table 1:** Baseline characteristics of X-linked hypophosphataemia cases and matched controls with at least one and two years of database history prior to the index date

	One year		Two years	
	XLH cases *N* = 8 (%)	Controls *N* = 60 (%)	XLH cases *N* = 7 (%)	Controls *N* = 51 (%)
Median age at the index date (IQR) - years	8.3 (3.7–10.9)	6.4 (2.2–10.4)	10.2 (5.3–11.4)	6.4 (4.2–10.4)
Gender – *N* (%)				
Males	2 (25.0)	17 (28.3)	2 (28.6)	17 (33.3)
Females	6 (75.0)	43 (71.7)	5 (71.4)	34 (66.7)
Physical characteristics^a^ - *N* (median [IQR])				
Height (m)	4 (1.1 [0.9–1.3])	30 (1.0 [0.8–1.2])	4 (1.2 [1.1–1.3])	27 (1.2 [0.9–1.3])
Weight (Kg)	5 (15.2 [14.5–27.6])	32 (15.8 [10.1–25.9])	5 (24.0 [15.2–27.6])	30 (21.7 [15.1–31.4])
BMI (Kg/m2)	4 (16.6 [16.2–17.2])	30 (17.3 [16.0–18.3])	4 (17.5 [16.5–18.6])	27 (17.6 [16.3–18.7])
Comorbidities^b^ - *N* (%)				
Diseases of oral cavity	1 (12.5)	10 (16.7)	1 (14.3)	10 (19.6)
Hearing loss	0 (0.0)	1 (1.7)	0 (0.0)	1 (2.0)
Fractures	1 (12.5)	2 (3.3)	1 (14.3)	2 (3.9)
Arthropathies and related disorders	0 (0.0)	1 (1.7)	0 (0.0)	1 (2.0)
Headache	1 (12.5)	2 (3.3)	1 (14.3)	2 (3.9)
Symptoms involving nervous and musculoskeletal systems	0 (0.0)	1 (1.7)	0 (0.0)	1 (2.0)
Malaise and fatigue	0 (0.0)	1 (1.7)	0 (0.0)	1 (2.0)
Asthma	2 (25.0)	7 (11.7)	2 (28.6)	7 (13.7)
Blindness and low vision	0 (0.0)	4 (6.7)	0 (0.0)	4 (7.8)
Lack of expected normal physiological development in childhood	0 (0.0)	0 (0.0)	0 (0.0)	0 (0.0)
Other acquired deformities of limbs	1 (12.5)	2 (3.3)	1 (14.3)	2 (3.9)
Drug prescriptions^c^ - Median [IQR]				
Number of individual drug prescriptions	3.0 [1.5–9.0]	1.0 [0.0–4.0]	8.0 [1.5–12.5]	3.0 [1.0–7.0]

*BMI* body mass index, *IQR* interquartile range, *XLH* X-linked hypophosphatemia

^a^Last available measurement registered prior to the index date

^b^Evaluated any time prior to the index date

^**c**^Evaluated one year prior to the index date

Statistically significant results from the univariable analysis are shown in Table 2. Concerning the one-year prior to ID analysis, as for continuous variables, the mean number of prescriptions of antihistamines for systemic use (ATC: R06*; mean ratio: 15.00, raw *p*-value = 0.003) and the prescription of hematological lab tests (mean ratio: 22.50; raw *p*-value = 0.017) were significantly higher among cases than controls. However, no statistically significant association persisted after the correction for multiple testing. Concerning the two-years prior to ID analysis, as for continuous variables, the mean number of vitamins prescription, mainly consisting of vitamin D and analogs (ATC: A11CC*; mean ratio: 480.86; raw *p*-value = 0.011), the mean number of antihistamines for systemic use prescriptions (ATC: R06*; mean ratio: 17.00; raw *p*-value < 0.001) and the mean number of pediatric follow-up visit or specialist visits requests (mean ratio: 2.97; raw *p*-value = 0.035) were significantly higher in cases than in controls. However, following the correction for multiple testing, the statistical significance persisted only for the number of prescriptions of antihistamines for systemic use (ATC: R06*; Bonferroni-corrected *p*-value = 0.009). As for the dichotomous predictors (i.e., comorbidities), the univariable analysis did not show any statistically significant association in both timeframes (data not shown).

**Table 2:** Total number of laboratory examinations and pharmacy claims (along with the mean number per patient), evaluated at one and two years prior to the index date, received by cases and matched controls included in the univariable analysis

Timeframe	Laboratory examination or pharmacy claims	Total number of registrations (mean number per patient)		Mean ratio^a^ (95% CI)	*p*-value (raw)^b^	Bonferroni-corrected *p*-value^b^
		XLH Cases (*N* = 8)	Controls (*N* = 60)			
One year prior to ID	Antihistamines for systemic use	4 (0.50)	2 (0.03)	15.00 (2.75–81.89)	0.003	0.108
	Hematological lab tests	3 (0.38)	1 (0.02)	22.50 (1.86–271.83)	0.017	0.510
Two years prior to ID	Vitamins	66 (9.43)	1 (0.02)	480.86 (4.93–46,855.11)	0.011	0.574
	Antihistamines for systemic use	7 (1.00)	3 (0.06)	17.00 (4.29–67.33)	<0.001	0.009
	Follow-up visit to FP/ specialist visits prescriptions	31 (4.43)	76 (1.49)	2.97 (1.11–7.98)	0.035	1.000

Only statistically significant results are shown

*CI* Confidence Interval, *FP* family pediatrician, *XLH* X-linked hypophosphatemia

^a^This measure quantifies how many times the average number of registrations per patient in XLH cases is higher than in matched controls. This measure was estimated by an over dispersed Poisson model, along with its 95% CI

^b^The “raw *p*-value” refers to the significance of the mean ratio, whereas the “Bonferroni-corrected *p*-value” corresponds to the raw *p*-value but corrected for multiple testing (i.e., inflated by a factor that controls the familywise error rate due to multiple hypothesis testing), according to the Bonferroni’s method

**Fig. 3:**
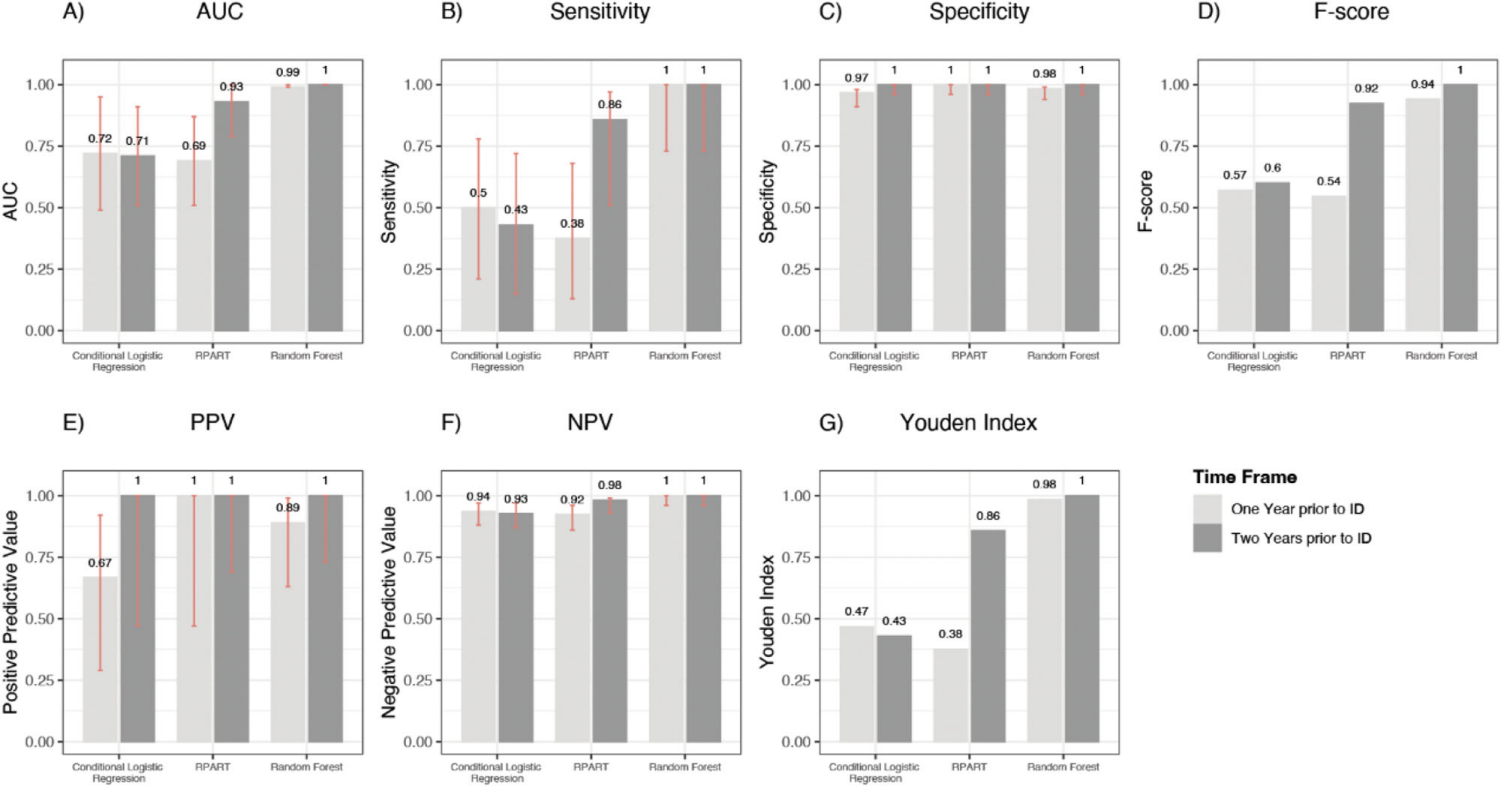
Performance of models for X-linked hypophosphatemia diagnosis prediction, both within 1 year and 2 years prior to the index date. **A** Area under the curve of the developed algorithms; **B** sensitivity of the developed algorithms; **C** specificity of the developed algorithms; **D** *F*-score of the developed algorithms; **E** positive predictive value of the developed algorithms; **F** negative predictive value of the developed algorithms; **G** Youden Index of the developed algorithms.

The performance of each proposed method (i.e., the conditional LASSO logistic model or machine-learning algorithms) in both the one-year and two-years prior ID analyses is shown in Fig. 3. Further input data regarding tuning parameters and optimal thresholds for each predictive model are shown in Supplementary Table 2.

The full list of variables selected by each model and the classification rules, or the relative variable importance (RVIMP) ranking, are shown in Table 3. Concerning the one-year prior ID analysis, the cross-validated multivariable conditional LASSO logistic model identified the number of vitamin prescriptions (mainly consisting of vitamin D and analogs) and the number of recorded diagnoses for acute respiratory infections as XLH predictors, achieving a fair degree of accuracy in discriminating patients from controls (AUC = 0.72). The XLH predictor selected by the RPART model was the number of vitamins prescriptions, achieving a lower degree of accuracy in discriminating patients from controls (AUC = 0.69). Finally, according to the Random Forest, the covariates with the RVIMP > 10% were the number of vitamins prescriptions, the number of antihistamines for systemic use prescriptions, the number of X-rays of the lower limbs and pelvis, and the number of allergology visit requests (AUC = 0.99). Concerning the two-years prior to ID analysis, the cross-validated multivariable conditional logistic regression with LASSO penalty model selected the number of vitamins prescriptions as XLH predictor, achieving a fair degree of accuracy in discriminating patients from controls (AUC = 0.71). The predictors selected by the RPART model were the number of vitamins prescriptions, the number of antihistamines for systemic use prescriptions and the prescription of the test for body composition assessment, achieving a high degree of accuracy in discriminating patients from controls (AUC = 0.93). Finally, concerning the Random Forest, the covariates with RVIMP > 10% were the number of vitamins prescriptions, the number of antihistamines for systemic use prescriptions and the prescription of X-ray of the lower limbs and pelvis (AUC = 1.00).

**Table 3:** List of predictors selected by each model (algorithm), along with the classification rule (or relative variable importance) to predict the presence of X-linked hypophosphataemia, among subjects with at least one and two years of database history prior to the index date




*ATC* anatomic, therapeutic, chemical classification system, ID index date, *IQR* interquartile range, *RVIMP* relative variable importance from random forest, *SD* standard deviation, *XLH* X-linked hypophosphataemia

^a^For both XLH cases and controls, this consists mainly of vitamin D and analogs (ATC: A11CC)

## Discussion

To our knowledge, this is the first population-based study that applied statistical models and machine learning algorithms to identify a combination of predictive variables for the early diagnosis of XLH using a pediatric general practitioners database.

The primary endpoint of the study, that is the annual prevalence of XLH cases in the PediaNet database, has been estimated to be 1.78 cases per 100,000 registered patients. XLH epidemiologic data reported in literature are poor and sometimes contradictory depending on the location of the epidemiologic evaluation; in Europe according to a Danish, a Norwegian and a French investigations, XLH prevalence can range from 1.07 to 4.8 cases per 100,000 inhabitants [4, 5, 28]; these data are in line with prevalence PediaNet findings.

The three models used to predict XLH diagnosis achieved a fair diagnostic accuracy, with random forest yielding the highest AUC values in both the one-year and the two-years prior to ID analyses. Overall, considering the one-year and the two-years prior to ID analyses together, six diagnosis predictors were selected by the three predictive algorithms. The selected predictors of XLH diagnosis were factors associated with the number of vitamin D prescriptions, mainly consisting of vitamin D and analogs, the number of recorded diagnoses for acute respiratory infections, the number of antihistamines for systemic use prescriptions, and the number of allergology visit prescriptions; other selected predictors of XLH diagnosis were specific exams or diagnostic tests (i.e., the number of X-rays of the lower limbs and pelvis, and the number of examinations for the body composition assessment). Regarding the number of vitamin D prescriptions, mainly consisting of vitamin D and analogs, it is reasonable that patients affected by hypophosphatemic hereditary disorders are usually treated with conventional therapy including active vitamin D and analogs to sustain inappropriately low vitamin D (1,25OH)2D3 levels. To date, the evidence concerning the potential association between vitamin D deficiency and acute respiratory infections is controversial. A large body of evidence suggests that vitamin D deficiency may be associated with increased autoimmunity and increased susceptibility to acute respiratory infections, including bronchiolitis and pneumonia, especially among children with vitamin D deficiency rickets [29–33]. In our study acute respiratory infections included acute nasopharyngitis, acute bronchitis and bronchiolitis, acute upper respiratory infections of multiple or unspecified sites, acute pharyngitis, acute bronchospasm, acute laryngitis and tracheitis, acute sinusitis and acute tonsillitis. In particular, a meta-analysis of eight observational studies exploring the association between serum vitamin D levels and community-acquired pneumonia (CAP) found that patients with vitamin D deficiency are at increased risk of CAP as compared to patients with normal vitamin D serum levels [34]. According to this meta-analysis, vitamin D is involved in the onset of CAP due to the binding of the active form of vitamin D (1,25OH)2D3, which is deficient in patients with XLH, to its receptor (VDR), which stimulates the expression of antibacterial peptides resisting bacterial and viral infections; moreover, it has been demonstrated that in in vivo experimental models, vitamin D deficiency causes the reduction of the level of VDR, thus damaging the epithelia of the respiratory tract mucous membranes with uncontrolled inflammatory reactions [34]. However, a clinical trial evaluating the association between vitamin D supplements and tuberculosis prevention did not find any benefit of vitamin D supplementation for the prevention of acute respiratory infection in children harboring severe vitamin D deficiency [35]. Therefore, patients included in our study may have an increased susceptibility to respiratory infections irrespective of vitamin D levels, as observed in other rare bone diseases such as the type 1 osteogenesis imperfecta [36–38].

Findings from our study showed that the number of allergology visit prescriptions as well as the number of antihistamines for systemic use prescriptions are two potential predictors of XLH diagnosis. This may be explained by the increasing, but still controversial, evidence demonstrating that low vitamin D levels are associated with an increased incidence of allergy, such as food allergy [39, 40] and allergic asthma [41, 42]. A recently published systematic review and meta-analysis of observational studies comparing vitamin D levels between children with food allergy and healthy controls reported that the first were found to have a 68% increased probability to experiment a food allergy episode, particularly in their second year of life, as well as a 56% increased probability of developing food sensitization [39].

It is indeed reported that vitamin D plays a crucial role in the modulation of the innate immune response, mainly by increasing the production of antimicrobial peptides (e.g., cathelicidin and β-defensin), as well as the adaptive immune response [43]. Since immune cells such as B cells, T cells, macrophages and dendritic cells express vitamin D receptors on their surfaces and they are capable of synthesizing active vitamin D, they can rapidly increase local levels of vitamin D, thus modulating adaptive immune responses [44]. Vitamin D inhibits B-cell proliferation, blocks B-cell differentiation and immunoglobulin secretion, suppresses T-cell proliferation and affects T-cell maturation, decreasing inflammatory cytokines such as interleukin (IL)-17 and IL-21 and increasing anti-inflammatory ones, such as IL-10 [43]. Moreover, vitamin D reduces mast cell activation, eosinophil count and infiltration of lung disease. These mechanisms, by reducing inflammation and modulating immune cells that are involved in the pathogenesis of allergic asthma, could explain the increased incidence of this pathology in rickets affected patients with inappropriately low active vitamin D levels.

The typical presentation of patients with XLH includes deformities of the lower limbs, bone pain, stunted growth, physical dysfunction and an increased prevalence of overweight/obesity in comparison to general population [10, 25]. Consequently, as expected, the other two XLH diagnosis predictors were related to XLH diagnostic procedures, i.e., the number of X-rays of the lower limbs and pelvis and the number of examinations for the body composition assessment.

One of the major strengths of this study is that data were collected from PediaNet, which is a large and validated research database managed by a national network of FPs, containing information on diagnoses, prescriptions, and outcomes for more than 430,000 children aged from 0 to 14 years of age during the study period. The size of the database and the long follow-up time are particularly relevant for rare diseases research, considering that the number of affected patients is very small. An additional strength is that cases were validated through manual search for FPs diagnosis in the clinical charts.

However, this study also has some limitations. First, a limited number of patients affected by XLH was found in PediaNet, and some of them did not have a sufficiently long database history, thus reducing the statistical power of the analyses. Second, many variables such as weight, height, head circumference, specialist visit (e.g., dentistry data) code and related free text report, but mostly clinical laboratory DMR alphanumeric code and analyte exam results, e.g., phosphate or vitamin D levels, were missing and information on hospitalizations, immunizations and privately purchased medications might be underreported, thus globally affecting the prediction analysis. Third, as the date of the first XLH diagnosis registered in PediaNet may not exactly coincide with the actual onset of the disease, the index date used to set the timeframes for diagnostic prediction might have been misclassified. Fourth, the exact indication of use of prescribed drugs, including vitamin D, was not available in PediaNet DB. Fifth, when available, anthropometric data may be affected by a measurement bias. Lastly, the predictor-diagnosis relationships assessed using data driven approaches, such as machine learning algorithms, do not always imply a causal relationship.

## Conclusion

In this study we internally evaluated the performance of algorithms for the prodromal diagnosis of XLH using a pediatric general practitioners’ research database. Findings showed that data-driven machine learning models may play a prominent role for the prediction of the diagnosis of rare diseases such as XLH. According to the three predictive models developed, the predictors significantly associated with the presence of XLH were the number of vitamin D and analogs prescriptions, the number of recorded diagnoses for acute respiratory infections, the number of antihistamines for systemic use prescriptions, the number of allergology visit prescriptions, the number of X-rays of the lower limbs and pelvis and the number of examinations for the body composition assessment.

### Supplementary information

Supplementary Table 1

Supplementary Table 2
