# Supplementary material for: Epidemiological analysis to identify predictors of X-linked hypophosphatemia (XLH) diagnosis in an Italian pediatric population: the EPIX project
Source: Endocrine. 2024 Apr 9;85(2):894–905. doi: 10.1007/s12020-024-03793-5 (PMC11291596; doi:10.1007/s12020-024-03793-5)
Supplement: Supplementary file 1 — Supplementary Table 1 [file 12020_2024_3793_MOESM1_ESM.docx]

**Epidemiological analysis to identify predictors of X-linked hypophosphatemia (XLH) diagnosis in an Italian pediatric population: the EPIX Project**

Salvatore Crisafulli^1*^, Ylenia Ingrasciotta^2*^, Giacomo Vitturi^2^, Andrea Fontana^3^, Luca L’Abbate^4^, Ylenia Alessi^5^, Francesco Ferraù^5^, Luigi Cantarutti^6^, Debora Lazzerini^7^, Salvatore Cannavò^5^, Gianluca Trifirò^2^

1. Department of Medicine, University of Verona, Verona, Italy
2. Department of Diagnostics and Public Health, University of Verona, Verona, Italy
3. Unit of Biostatistics, IRCCS Casa Sollievo della Sofferenza, Foggia, Italy
4. Department of Biomedical and Dental Sciences and Morphofunctional Imaging, University of Messina, Messina, Italy
5. Department of Human Pathology of Adulthood and Childhood "G. Barresi" DETEV, University of Messina, Messina, Italy
6. Società Servizi Telematici – Pedianet Project, Padova, Italy
7. Medical Affairs, Kyowa Kyrin, Milano, Italy

**Supplementary Table 1.** Data sources and list of variables (name, type and format)

| **Dataset** | **Information** |
| --- | --- |
| **Pediatrician visits dataset** | - Pediatrician ID (numeric code)  - Patient ID (alphanumeric code)  - Visit date (dd/mm/yyyy)  - Visit time (hh:mm)  - Visit reason (free text)  - Diagnosis text (free text)  - Diagnosis ICD-9 code (alphanumeric code)*  - Signs and symptoms text (free text)  - Signs and symptoms ICD-9 code (alphanumeric code)* |
| **Co-payment exemptions dataset** | - Pediatrician ID (numeric code)  - Patient ID (alphanumeric code)  - Exemption code (alphanumeric code)  - Exemption description (free text)  - Start exemption date (dd/mm/yyyy)  - End exemption date (dd/mm/yyyy) |
| **Drug prescriptions dataset** | - Pediatrician ID (numeric code)  - Patient ID (alphanumeric code)  - Date of prescription (dd/mm/yyyy)  - Drug AIC code (alphanumeric code)  - Drug description (free text)  - Active substance (free text)  - Number of dispensed drugs (numeric, counts)  - Drug price (numeric, Euro)  - Drug ATC code (alphanumeric code) |
| **Physical characteristics (measures) dataset** | - Pediatrician ID (numeric code)  - Patient ID (alphanumeric code)  - Date of birth (dd/mm/yyy)  - Gender (character)  - Visit date (dd/mm/yyyy)  - Weight (numeric, Kg)  - Height (numeric, cm)*  - Head circumference (numeric, cm)* |
| **Specialist examinations^#^ dataset** | - Pediatrician ID (numeric code)  - Patient ID (alphanumeric code)  - Receipt ID (alphanumeric code)  - Receipt date (dd/mm/yyyy)  - Specialist exam DMR (alphanumeric code)*  - Specialist exam description (free text)  - Branch ID (numeric code)  - Report (free text)* |
| **Laboratory exams dataset** | - Pediatrician ID (numeric code)  - Patient ID (alphanumeric code)  - Receipt ID (alphanumeric code)  - Receipt date (dd/mm/yyyy)  - Lab test description (free text)  - Lab DMR (alphanumeric code)*  - Branch ID (numeric code)  - Exam cost (numeric, Euro) |
| **Clinical lab analytes dataset** | - Pediatrician ID (numeric code)  - Patient ID (alphanumeric code)  - Laboratory ID (alphanumeric code)  - Receipt ID (alphanumeric code)  - Receipt date (dd/mm/yyyy)  - Report ID code (numeric code)  - Analyte exam description (free text)  - Analyte exam result (free text)* |

*this variable includes many missing information; ^#^diagnostic tests and specialist visits
