# Supplementary material for: Epidemiological analysis to identify predictors of X-linked hypophosphatemia (XLH) diagnosis in an Italian pediatric population: the EPIX project
Source: Endocrine. 2024 Apr 9;85(2):894–905. doi: 10.1007/s12020-024-03793-5 (PMC11291596; doi:10.1007/s12020-024-03793-5)
Supplement: Supplementary file 2 — Supplementary Table 2 [file 12020_2024_3793_MOESM2_ESM.docx]

**Epidemiological analysis to identify predictors of X-linked hypophosphatemia (XLH) diagnosis in an Italian pediatric population: the EPIX Project**

Salvatore Crisafulli^1*^, Ylenia Ingrasciotta^2*^, Giacomo Vitturi^2^, Andrea Fontana^3^, Luca L’Abbate^4^, Ylenia Alessi^5^, Francesco Ferraù^5^, Luigi Cantarutti^6^, Debora Lazzerini^7^, Salvatore Cannavò^5^, Gianluca Trifirò^2^

1. Department of Medicine, University of Verona, Verona, Italy
2. Department of Diagnostics and Public Health, University of Verona, Verona, Italy
3. Unit of Biostatistics, IRCCS Casa Sollievo della Sofferenza, Foggia, Italy
4. Department of Biomedical and Dental Sciences and Morphofunctional Imaging, University of Messina, Messina, Italy
5. Department of Human Pathology of Adulthood and Childhood "G. Barresi" DETEV, University of Messina, Messina, Italy
6. Società Servizi Telematici – Pedianet Project, Padova, Italy
7. Medical Affairs, Kyowa Kyrin, Milano, Italy

**Supplementary Table 2.** Summary of predictive models (algorithms) performance evaluated within one and two years prior to the index date.

| **Timeframe** | **Model name** | **Model output** | **Optimal tuning**  **parameters** | **N. of selected covariates** | **Optimal threshold** | **AUC (95%CI)** | **SE** | **SP** | **PPV** | **NPV** | **Youden Index** | **F-score** |
| --- | --- | --- | --- | --- | --- | --- | --- | --- | --- | --- | --- | --- |
| One year prior to ID | Cross-Validated multivariable conditional logistic regression with LASSO penalty | Binary  classification (based on optimal  threshold of model’s linear predictor) | Penalty parameter  = 3.299 | N=2 | 0.072 | 0.72  (0.49-0.95) | 0.500 | 0.967 | 0.667 | 0.935 | 0.467 | 0.571 |
|  | Classification tree defined by the RPART algorithm | Individual probabilities | Nodesize= 3  (CP=0.125) | N=1 | 0.538 | 0.69  (0.51-0.87) | 0.375 | 1.000 | 1.000 | 0.923 | 0.375 | 0.545 |
|  | Random Forest | Individual probabilities | N. trees=100,000;  Nodesize=15 | N=4* | 0.248 | 0.99  (0.99-1.00) | 1.000 | 0.983 | 0.889 | 1.000 | 0.983 | 0.941 |
| Two years prior to ID | Cross-Validated multivariable conditional logistic regression with LASSO penalty | Binary  classification (based on optimal  threshold of model’s linear predictor) | Penalty parameter = 6.777 | N=1 | 0.892 | 0.71  (0.51-0.91) | 0.429 | 1.000 | 1.000 | 0.927 | 0.429 | 0.600 |
|  | Classification tree defined by the RPART algorithm | Individual probabilities | Nodesize= 3  (CP=0.143) | N=3 | 0.510 | 0.93  (0.79-1.00) | 0.857 | 1.000 | 1.000 | 0.981 | 0.857 | 0.923 |
|  | Random Forest | Individual probabilities | N. trees=100,000;  Nodesize=15 | N=3* | 0.214 | 1.00  (1.00 -1.00) | 1.000 | 1.000 | 1.000 | 1.000 | 1.000 | 1.000 |

**Abbreviations**: AUC: Area Under the Receiver Operator Characteristic (ROC) Curve on the estimated individual probabilities; CI: confidence interval computed using the DeLong method; RPART: Recursive PArtitioning and Regression Tree; CP: complexity parameter needed to prune the large tree; N. trees: Number of trees included in the Random Forest; Nodesize: minimum size of terminal nodes for each tree defined in the forest; ID: index date; SE: Sensitivity (i.e., recall); SP: Specificity; PPV: Positive Predictive Value; NPV: Negative Predictive Value;

**Notes**:

1. SE, SP, PPV, NPV and F-score were estimated at the “optimal threshold” of the individual estimated probabilities (i.e., the one that reached the highest Youden Index in the ROC curve);
2. Since Random Forest uses all candidate predictors to estimate the probability of XLH, it is obvious to expect its AUC and diagnostic measures to be the highest (AUC=1)

*Number of predictors that achieved a relative Variable Importance > 10% from the Random Forest algorithm.
